# Asthma-Specific Temporal Variability Reveals the Effect of Group Cognitive Behavior Therapy in Asthmatic Patients

**DOI:** 10.3389/fneur.2021.615820

**Published:** 2021-03-12

**Authors:** Yuqun Zhang, Youyong Kong, Yuan Yang, Yingyin Yin, Zhenghua Hou, Zhi Xu, Yonggui Yuan

**Affiliations:** ^1^School of Nursing, Nanjing University of Chinese Medicine, Nanjing, China; ^2^Department of Psychosomatic and Psychiatry, Zhongda Hospital, School of Medicine, Southeast University, Nanjing, China; ^3^Lab of Image Science and Technology, Key Laboratory of Computer Network and Information Integration, School of Computer Science and Engineering, Ministry of Education, Southeast University, Nanjing, China; ^4^Department of Respiratory, Zhongda Hospital, School of Medicine, Southeast University, Nanjing, China

**Keywords:** asthma, group cognitive behavior therapy, functional connectivity, temporal variability, neuroimaging biomarker

## Abstract

**Background:** Group cognitive behavior therapy (GCBT) is a successful therapy for asthma. However, the neural biomarker of GCBT which could be used in clinic remains unclear. The temporal variability is a novel concept to characterize the dynamic functional connectivity (FC), which has many advantages as biomarker. Therefore, the aim of this study is to explore the potential difference of temporal variability between asthmatic patients and healthy controls, then determine the different patterns of temporal variability between pre- and post-treatment group and reveal the relationship between the variability and the symptoms improvement reduced by GCBT.

**Methods:** At baseline, 40 asthmatic patients and 40 matched controls received resting-state functional magnetic resonance imaging (fMRI) scans and clinical assessments. After 8 weeks of GCBT treatment, 17 patients received fMRI scans, and assessments again. Temporal variability at baseline and post-treatment were calculated for further analysis.

**Results:** Compared with controls, asthmatic patients showed widespread decreases in temporal variability. Moreover, the variability in both right caudate and left putamen were positively correlated with asthma control level. After GCBT, asthma control level and depression of patients were improved. Meanwhile, compared with pre-GCBT, patients after treatment showed lower variability in left opercular of Rolandic, right parahippocampal gyrus and right lingual gyrus, as well as higher variability in left temporal pole. Variability in regions which were found abnormal at baseline did not exhibit significant differences between post-GCBT and controls.

**Conclusions:** Asthma-specific changes of dynamic functional connectivity may serve as promising underpinnings of GCBT for asthma.

**Clinical Trial Registration:** http://www.chictr.org.cn/index.aspx, identifier: Chi-CTR-15007442.

## Introduction

Asthma is a common disorder with a high prevalence of psychiatric disorders (1). There are almost 300 million asthmatic patients all over the world to the end of 2012 (2). The increasing trends may be associated with the changes in environment pollutions (3), lifestyle and rapid urbanization (1). Moreover, psychiatric comorbidities are increasingly recognized as important determinants of asthma management and prognosis, as they are involved in undesirable disease control and poor quality of life (4–6). The highest prevalence, comorbidities and bad managements contribute to high costs for the society. Given that even small reductions in psychiatric comorbidities and small improvements in asthma management have a greater public health benefit than treating those who are already symptomatic or always recurring, more effort should be targeted toward developing, evaluating, and implementing interventions for asthma-related education and negative emotions.

Recent research consistently demonstrated that group cognitive behavior therapy (GCBT) can encourage asthmatic patients to accept their problems, keep control of their symptoms and medications, and alleviate asthma-related emotional symptoms (7–9). Our previous studies demonstrated that GCBT is a successful psychotherapy in improving the asthma-related symptoms (10, 11). However, the therapeutic mechanism of GCBT remains unclear. Magnetic resonance imaging (MRI) techniques provide the opportunities to investigate the neural underpinnings of psychotherapy. Previous studies reported the recovered effects of GCBT for asthma (10, 11), depression (12, 13), and anxiety (14–16), while consistent findings are not confirmed.

Temporal variability is a novel concept to quantitatively characterize the dynamic functional connectivity (FC) from the blood oxygen level-dependent (BOLD) signals (17). This metric can obtain the stability or flexibility of the regional network within a specific time window. Accumulating evidence indicates that temporal variability can be used to explain intra-subject variability in diseases and considered as a biomarker related to treatment response (18, 19). For example, Huang et al. (18) observed a global reduction in the temporal variability, local, and distant brain signal synchronization for subjects during anesthesia. Hou et al. (19) found that increased temporal variability of the striatum region predicted early antidepressant response in patients with major depressive disorder. As alluded to before, resting FC in GCBT has been studied widely in recent years (10, 11), but similar research on dynamic FC has been quite scarce.

Accordingly, to find the effective neuroimaging biomarkers of GCBT which can be used in clinic, the aim of this study was to find the asthma specific features of temporal variability by analyzing the BOLD data of asthmatic patients and healthy controls, then determine the different patterns of temporal variability between pre- and post-GCBT patients and reveal the relationships between variability and clinical symptoms changed by GCBT. We hypothesized that asthmatic patients might show distinctive temporal variability characteristics, and GCBT could improve the clinical symptoms by regulating abnormal temporal variability.

## Methods and Materials

### Participants and Evaluations

Forty patients with a diagnosis of bronchial asthma without acute attacks and 40 age-, gender- and education level-matched healthy controls (HC) were recruited. All participants underwent an MRI scan with the same imaging parameter. Patients also received a series of clinical assessments including a 17-item Hamilton depression rating scale (HAMD), Chinese-version short health anxiety inventory (CSHAI) and asthma control test (ACT) (Table 1). In addition, there were 17 patients who completed GCBT treatment. They also received the above assessments after GCBT. The contents of GCBT were reported in our previous study, which consisted of 8 sessions (10). This study was approved by the recommendations of the ethics committee (Zhongda Hospital, Southeast University, Nanjing, China, No. 2016ZDSYLL004.0) with written informed consent from all subjects. It is in accordance with the declaration of Helsinki. The clinical trial registration number is Chi-CTR-15007442.

**Table 1:** The demographics and clinical scales.

**Characteristics**		**Asthma (*N =* 40)**	**HC (*N =* 40)**	***P-*value**
Age (years)		51.4 ± 9.93	50.83 ± 9.04	0.787
Gender (male)		17	14	0.491^a^
Education (years)		11.95 ± 2.56	11.23 ± 2.64	0.216
ACT		17.53 ± 4.81	NA	
CSHAI	Total scores	13.45 ± 6.40	NA	
	IL	10.35 ± 5.41	NA	
	NC	2.90 ± 2.30	NA	
HAMD		6.13 ± 5.48	0.93 ± 1.12	<0.001
		**Pre-GCBT (*****N =*** **17)**	**Post-GCBT (*****N =*** **17)**	***P-*****value**
ACT		16.471 ± 4.27	21.06 ± 4.28	0.001
	Total scores	13.88 ± 7.77	12.56 ± 5.63	0.285
CSHAI	IL	11.29 ± 5.84	10.06 ± 4.93	0.368
	NC	2.58 ± 2.45	2.00 ± 1.66	0.289
HAMD		5.59 ± 5.40	1.82 ± 2.35	<0.001
		**HC (*****N =*** **40)**	**Post-GCBT (*****N =*** **17)**	***P-*****value**
Age (years)		50.83 ± 9.04	50.76 ± 12.15	0.984
Gender (male)		14	7	0.658 ^a^
Education (years)		11.23 ± 2.64	11.12 ± 2.83	0.257
HAMD		0.93 ± 1.12	1.82 ± 2.35	0.055

*Data are expressed as mean ± standard deviation*,

a *Chi-square test*.

*HC, healthy controls; ACT, asthma control test; CSHAI, Chinese-version short healthy anxiety inventory; HAMD, Hamilton depression rating scale; GCBT, group cognitive behavior therapy*.

Participants were all right-handed and had at least 6 years of education, with ages ranging from 18 to 65 years. They had no electronic and metal equipment in their bodies (such as cardiac pacemaker, defibrillator, stent). Patients met the diagnostic criteria of bronchial asthma and during non-attacks. Participants were excluded if (1) they suffered from other serious disease of respiratory system; (2) presented with mental disorders and alcohol and drug dependence; (3) had a history of organic brain disorders and cardio, hepatic, renal abnormality; (4) were women during pregnancy or lactation.

### Brain Image Acquisition

MRI studies were performed on a 3-Tesla Scanner (Siemens, Erlangen, Germany) using a homogeneous birdcage head coil. All the subjects were scanned with high resolutional three dimensional T1 weighted imaging and resting state fMRI. The T1 weighted images were obtained using magnetization prepared rapid gradient echo sequences with the following parameters: repetition time (TR) = 1,900 ms, echo time (TE) = 2.48 ms, flip angle (FA) = 9°, acquisition matrix = 256 × 256, field of view (FOV) = 250 × 250 mm^2^, thickness = 1.0 mm, gap = 0, slice = 176. An 8 min resting state fMRI was acquired with using a gradient -recalled echo-planar imaging pulse sequence the following parameters: TR = 2,000 ms, TE = 25 ms, FA = 90°, acquisition matrix = 64 × 64; FOV = 240 × 240 mm^2^; thickness = 3.0 mm; gap = 0 mm; 36 axial slices; 240 volumes; 3.75 × 3.75 mm^2^ in-plane resolution parallel to the anterior-posterior commissure line. Participants lay supine with the head snugly fixed by a belt and foam pads to minimize head motion, and were required to keep their eyes closed, stay awake, and not think of specific things during scanning.

### Functional Imaging Preprocessing

All the resting state fMRI data were preprocessed using the Data Processing Assistant for Resting-State Function (DPARSF 2.3 Advanced Edition) toolkit. For each subject, the first ten frames were discarded for magnetic saturation. The following steps were performed: (1) slice timing correction; (2) motion correction; (3) co-registering T1 to functional image; (4) spatial normalization to Montreal Neurological Institute space; (5) spatial smoothing using a 6 mm full-width at half-maximum Gaussian kernel; (6) linear detrend; (7) regression of nuisance signals (white matter, cerebrospinal fluid signals and global signal), and the head-motion parameters; (8) temporal band-passing (0.01~0.08 Hz) to minimize low-frequency drift and filtering the high-frequency noise.

### Temporal Variability of Functional Network

**Figure 1:**
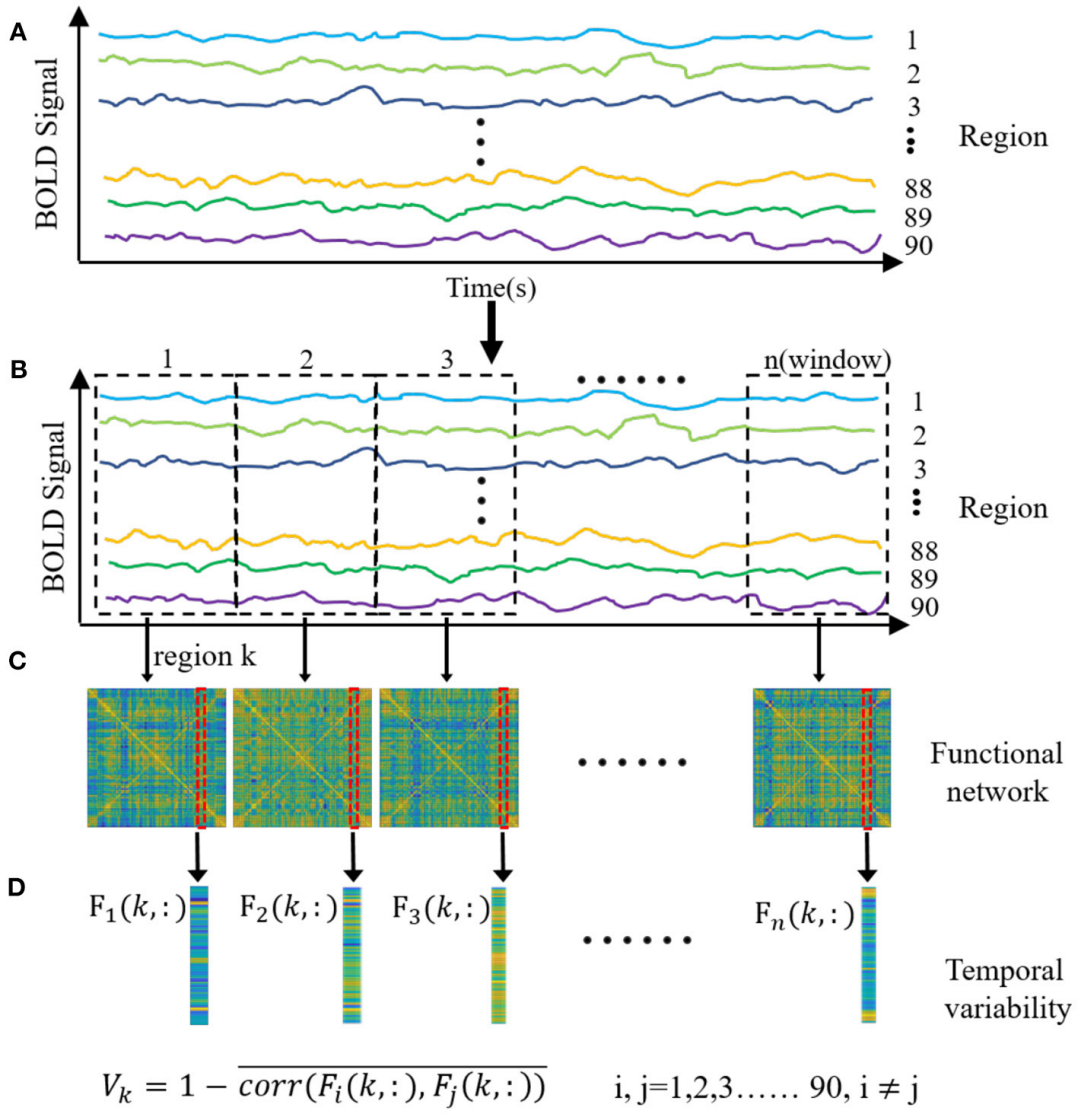
The main procedure of the temporal variability analysis. **(A)** The time courses were averaged in each of brain regions with the anatomical automatic labeling atlas-90. **(B)** The entire time courses were segmented into several non-overlapping windows with a specific temporal length. **(C)** Functional connectivity was obtained for each window by computing Pearson correlation between each brain region. **(D)** Temporal variability was obtained for each brain region by calculating the variability of the functional architecture. The functional architecture of a brain region k at a time window n is defined as the overall functional connectivity between current region k and other regions. The temporal variability of the region k is 1 minus the mean values of correlations of functional architectures among different windows for the current region.

Temporal variability was performed to capture the dynamic functional network for all the subjects from the patients and controls. The main procedure of the temporal variability calculation is illustrated in Figure 1. First of all, time courses were averaged in each of brain regions with the selected parcellation, shown as Figure 1A. The anatomical automatic labeling atlas-90 (AAL-90) was utilized to parcellate the brain, which comprised of 45 cortical and subcortical nodes (i.e., brain regions) in each hemisphere. Secondly, the entire time courses were segmented into several non-overlapping windows with a specific temporal length, shown as Figure 1B. The length of window was set ranging from 42 s to 60 s, which has been demonstrated to obtain robust brain connectivity results, and to sufficiently capture the dynamic features (20). Thirdly, for each subject, functional connectivity was obtained for each window by computing Pearson correlation between each brain region, shown as Figure 1C. Finally, temporal variability was obtained for each brain region by calculating the variability of the functional architecture, shown as Figure 1D. The functional architecture of a brain region k at a time window n is defined as the overall functional connectivity between current region k and other regions. The temporal variability of a region k is 1 minus the mean values of correlations of functional architectures among different windows for the current region (17). The value of temporal variability can range from 0 to 2. A larger value represents a high temporal variability for the brain region. To avoid the arbitrary choice of window length, the temporal variability was calculated in different length of temporal windows, which ranged from 21 to 30 volumes. The final temporal variability was achieved by calculating the average values in different lengths of windows for each node of all the subjects. We can get a temporal variability value for each brain region of each subject. Therefore, a total of 90 temporal variability values was obtained for each subject in both patients and controls.

### Statistical Analysis

Predictive Analytics Software (PASW) Statistics 18 package (IBM Corporation, Armonk, NY, USA) was applied to complete the analyses. Independent two-sample *t*-test, Chi-square test, paired *t*-test and Pearson's correlation analysis were used to determine significant differences in demographic data, clinical scale scores, and temporal variability values. For comparisons of age, HAMD scores, ACT scores, CSHAI scores and temporal variability, differences between patients pre-GCBT and controls, as well as patients post-GCBT and controls were determined by two-sample *t*-test. The difference of gender was revealed by Chi-square test. The relationships between clinical features (depression severity, asthma control level, health anxiety level and symptoms reduction rate) and temporal variability were analyzed by Pearson's correlation analysis. *P* < 0.05 (uncorrected) was considered to indicate statistical significance.

### Intervention

The content of GCBT for asthma included eight sessions, with each session lasting ~60 min weekly. Each group consisted of 6–8 patients. The detailed treatment strategies are described in our previous study (10).

## Results

### Demographic and Clinical Data

The detailed demographic and clinical information were showed in Table 1. There were no significant differences of age, gender, and education between asthmatic patients and HC. The HAMD scores of patients were significantly higher than controls (*P* < 0.001). After GCBT treatment, ACT scores (*P* < 0.001) were higher than before, while HAMD scores were lower (*P* < 0.001). Moreover, no significant difference was found in HAMD scores between patients post-GCBT and controls (*P* = 0.055).

### Comparisons of Temporal Variability

**Figure 2:**
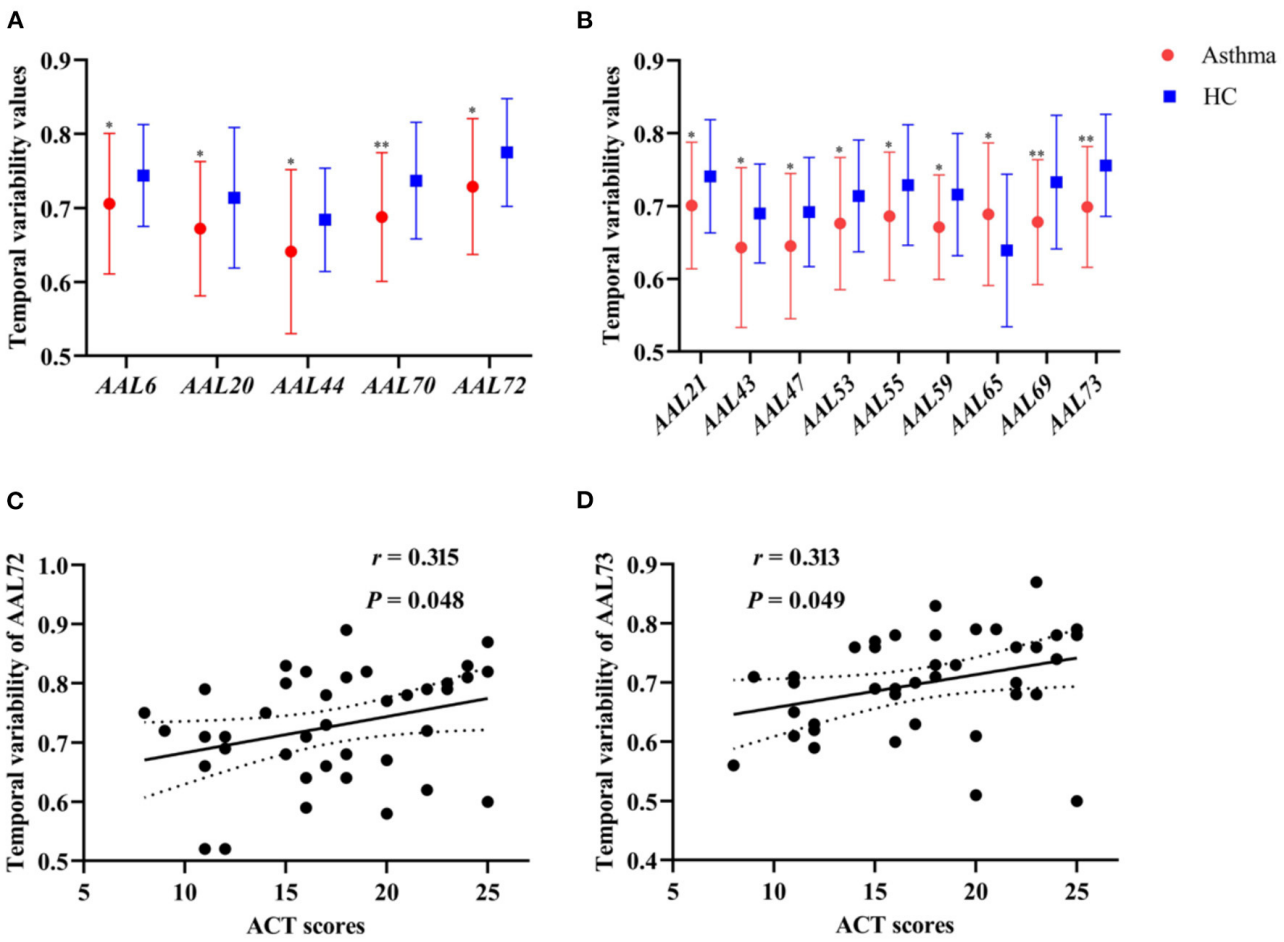
**(A,B)** The comparisons of temporal variability between asthmatic patients and controls in the right and left cerebral hemisphere, respectively (^*^P < 0.05, ^**^P < 0.01). **(C,D)** The correlation between temporal variability of AAL 72 (right caudate) and ACT scores (*r* = 0.315, *P* = 0.048), as well as temporal variability of AAL 73 (left putamen) and ACT scores (*r* = 0.313, *P* = 0.049) in asthmatic patients at baseline. HC, healthy controls; ACT, asthma control test.

To identify asthma-specific changes, a whole-brain temporal variability analysis was performed in asthmatic patients by comparing them to controls at baseline. We note that more than 15% of brain regions (*N* = 13) showed lower temporal variability in asthma (Figures 2A,B and Table 2) (all *P* < 0.05), including right orbital part of superior frontal (AAL 6), right supplemental motor area (SMA, AAL 20), left olfactory (AAL 21), left calcarine cortex (AAL 43), right calcarine (AAL 44), left lingual gyrus (AAL 47), left inferior occipital gyrus (AAL 53), left fusiform gyrus (AAL 55), left superior parietal gyrus (AAL 59), left paracentral lobule (AAL 69), right paracentral lobule (AAL70), right caudate (AAL 72) and left putamen (AAL 73). While variability increased mainly in left angular gyrus (AAL 65). When the functional architecture of a given region shows high correlation across different time windows, or the dynamical functional connectivity time series exhibit high synchronization between the region of interest and other regions, the temporal variability will be low (17). On the contrary, temporal variability will be high when the dynamical functional connectivity time series between the given region and other regions are independent (17).

**Table 2:** The comparisons of temporal variability between asthmatic patients and controls.

**Items**	**AAL**	**Side**	**Asthma (*N =* 40)**	**HC (*N =* 40)**	***P*-value**
**Asthma < HC**					
Orbital superior frontal gyrus	6	R	0.706 ± 0.095	0.744 ± 0.069	0.047
Supplemental motor area	20	R	0.672 ± 0.091	0.714 ± 0.095	0.050
Olfactory	21	L	0.701 ± 0.087	0.741 ± 0.078	0.032
Calcarine	43	L	0.643 ± 0.110	0.690 ± 0.068	0.025
Calcarine	44	R	0.641 ± 0.111	0.684 ± 0.070	0.040
Lingual	47	L	0.645 ± 0.100	0.692 ± 0.075	0.020
Inferior occipital gyrus	53	L	0.676 ± 0.091	0.714 ± 0.077	0.048
Fusiform	55	L	0.686 ± 0.088	0.729 ± 0.083	0.027
Superior parietal gyrus	59	L	0.671 ± 0.072	0.716 ± 0.084	0.013
Paracentral lobule	69	L	0.678 ± 0.086	0.733 ± 0.092	0.007
Paracentral lobule	70	R	0.688 ± 0.087	0.737 ± 0.079	0.010
Caudate	72	R	0.729 ± 0.092	0.775 ± 0.073	0.014
Putamen	73	L	0.699 ± 0.083	0.756 ± 0.070	0.002
**Asthma > HC**					
Angular	65	L	0.689 ± 0.098	0.639 ± 0.105	0.033

*Continuous variables are presented as mean ± standard deviation (SD). HC, healthy controls; R, right; L, left*.

**Figure 3:**
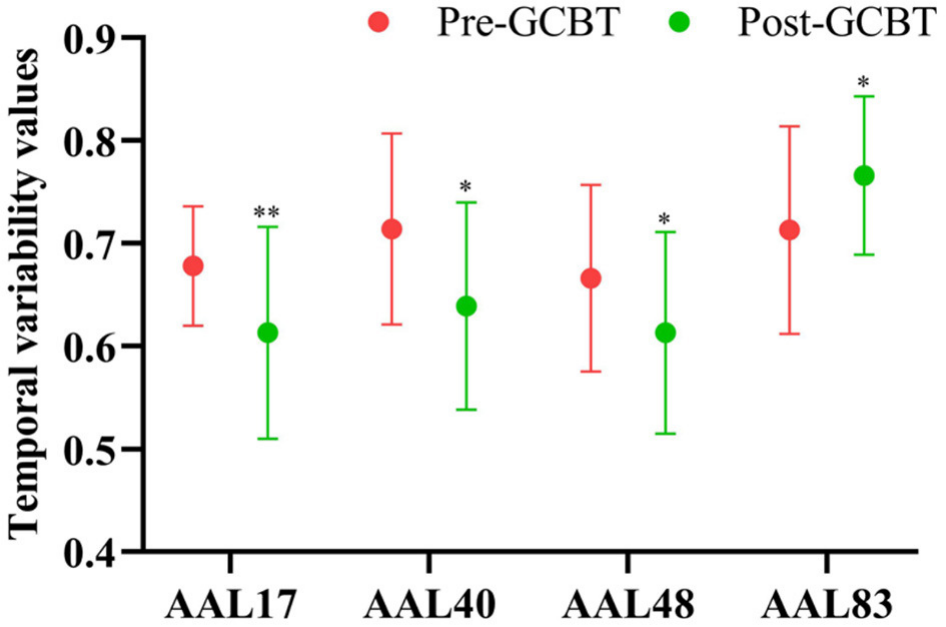
The comparisons of temporal variability between asthmatic patients pre- and post-GCBT (^*^P < 0.05, ^**^P < 0.01). GCBT, group cognitive behavior therapy.

Compared to pre-GCBT, patients after GCBT showed lower temporal variability in the left opercular of Rolandic (AAL 17) (*P* < 0.01), right parahippocampal gyrus (AAL 40) (*P* < 0.05), and right lingual gyrus (AAL 48) (*P* < 0.05). While higher temporal variability in the left temporal pole was also found in the patients after treatment (AAL 83) (*P* < 0.05) (Figure 3, Table 3).

**Table 3:** The comparisons of temporal variability between pre- and post-GCBT.

**Items**	**AAL**	**Side**	**Pre-GCBT (*N =* 17)**	**Post-GCBT (*N =* 17)**	***P*-value**
**Pre-GCBT > Post-GCBT**					
Rolandic operculum	17	L	0.678 ± 0.058	0.613 ± 0.103	0.008
Parahippocampal gyrus	40	R	0.714 ± 0.093	0.639 ± 0.101	0.017
Lingual gyrus	48	R	0.666 ± 0.091	0.613 ± 0.098	0.047
**Pre-GCBT < Post-GCBT**					
Temporal pole	83	L	0.713 ± 0.101	0.766 ± 0.077	0.049

*Continuous variables are presented as mean ± standard deviation (SD). GCBT, group cognitive behavior therapy; R, right; L, left*.

**Figure 4:**
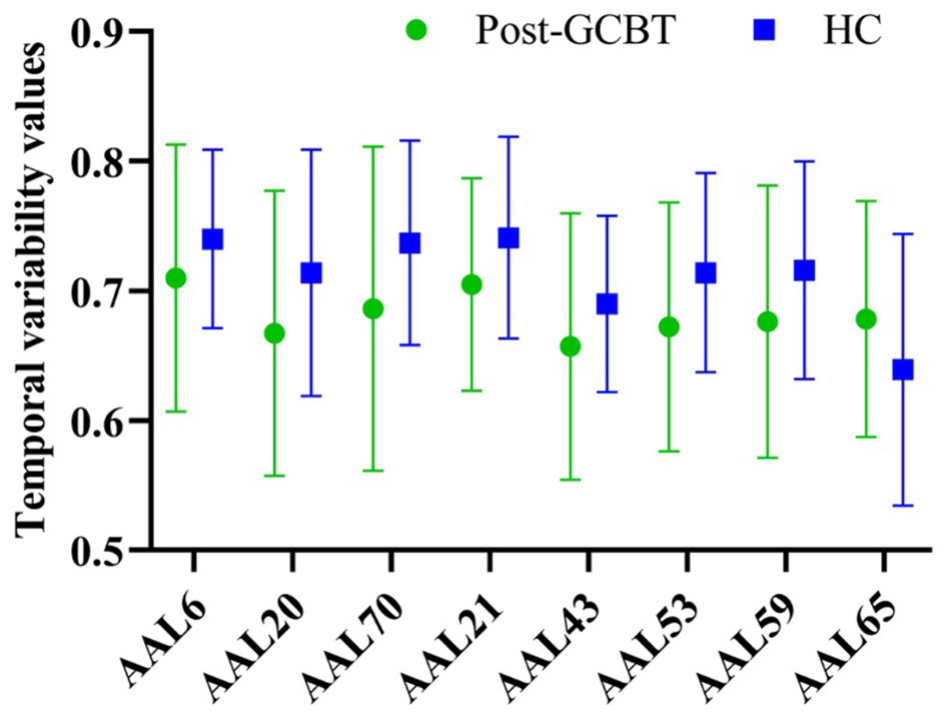
The comparisons of temporal variability between asthmatic patients post-GCBT and controls. GCBT, group cognitive behavior therapy; HC, healthy controls.

Furthermore, we compared the temporal variability between patients post-GCBT and HC. There was no significant difference in variability between them in the right orbital superior frontal gyrus (AAL 6), right SMA (AAL 20), left olfactory (AAL 21), left calcarine (AAL 43), left inferior occipital gyrus (AAL 53), left superior parietal gyrus (AAL 59), left angular (AAL 65), and right paracentral lobule (AAL 70) (Figure 4, Table 4). It means that GCBT may improve the clinical symptoms by reversing the variability in the above brain regions.

**Table 4:** Brain regions with no difference in temporal variability between post-GCBT and HC.

**Items**	**AAL**	**Side**	**Post-GCBT (*N =* 17)**	**HC (*N =* 40)**	***P*-value**
Orbital part of superior frontal gyrus	6	R	0.710 ± 0.103	0.744 ± 0.069	0.232
Supplemental motor area	20	R	0.667 ± 0.110	0.714 ± 0.095	0.108
Olfactory	21	L	0.705 ± 0.082	0.741 ± 0.078	0.119
Calcarine	43	L	0.657 ± 0.103	0.690 ± 0.068	0.162
Inferior occipital gyrus	53	L	0.672 ± 0.096	0.714 ± 0.077	0.086
Superior parietal gyrus	59	L	0.676 ± 0.105	0.716 ± 0.084	0.132
Angular	65	L	0.678 ± 0.091	0.639 ± 0.105	0.194
Paracentral lobule	70	R	0.686 ± 0.125	0.737 ± 0.079	0.068

*Continuous variables are presented as mean ± standard deviation (SD). HC, healthy controls; GCBT, group cognitive behavior therapy; SMA, supplemental motor area; R, right; L, left*.

### Correlations Between Temporal Variability and Clinical Symptoms

The temporal variability of right caudate (*r* = 0.315, *P* = 0.048, *uncorrected*) and left putamen (*r* = 0.313, *P* = 0.049, *uncorrected*) was significantly positively associated with ACT scores at baseline in asthmatic patients, respectively (Figures 2C,D). No statistically significant correlation was found between temporal variability and clinical symptom improvement (HAMD, ACT, and CSHAI reduction rate).

## Discussion

In this study, differences of temporal variability between asthmatic patients and controls, as well as between pre- and post-GCBT were comprehensively investigated. The clinical relevance of temporal variability was also examined. Compared to controls, asthmatic patients showed lower temporal variability in parts of orbitofrontal cortex (OFC), visual network and sensorimotor cortex, which were generally correlated with asthma control level. Notably, after GCBT, patients exhibited treatment-specific changes of variability in the regions which have no differences between asthma and controls at baseline.

### Asthma-Specific Changes of Variability

Previous studies of resting-state FC (10, 21, 22) and regional homogeneity (ReHo) (11) analysis have revealed changes in the intrinsic topographical organization of the brain in asthma. However, time-varying properties of the brain in asthma have not been investigated. This study detected asthma-specific changes of variability, which may provide the evidence to further understand the neuroimaging underpinnings of asthma.

The orbital part of the superior frontal gyrus is part of the OFC, which is involved in sensory integration (23). In terms of anatomical structure, the OFC connects with many critical cortices, including motor, sensory, and some regions of limbic system. The extensive connections between OFC and other regions indicated the complex functions of it (24). In 1987, Marks et al. (25) have demonstrated that OFC is involved in respiratory phase switching, and it influences the brain stem structures during rapid-eye-movement sleep. In line with this finding, asthmatic patients showed decreased variability in OFC. Therefore, we inferred that the lower variability in OFC would be also associated with the aberrant respiratory in asthma. Meanwhile, variability in visual network (i.e., calcarine, lingual gyrus and inferior occipital gyrus) of asthmatic patients was also lower than of controls. Physiologically, the visual cortex is associated with selective attention, which can be readily captured by salient emotional distractors (26). In particular, asthmatic patients tend to exhibit depressive emotion. These studies indicated that the decreased variability of the visual network may reflect the attentional biases of negative emotions in asthma. For example, Li et al. (27) have demonstrated that the widespread decreased regional function in visual network is the core change which is influenced by asthma. Thus, abnormal variability in the visual network may be an asthma-specific characteristic, especially the key changes involved in emotional biases in asthma.

It seems that breathing is so simple, yet taking a breath is a complex motor function, which is associated with the coordination of neural activation of many brain regions (28). Movement-activity was more frequent in the supplemental motor area (SMA), and its onset is often time locked to the movement onset (29). Kojima et al. (30) investigated the changes of hemoglobin levels in SMA during cardiopulmonary exercise test, and they found that total hemoglobin of SMA increased at respiratory compensation in healthy people. This means that the asthmatic patients with decompensated respiration would present abnormal function in SMA which is influenced by abnormal hemoglobin levels. As an important node of the motor network, the SMA is involved in the lower arterial oxygen saturation (31). Decreased FC between the right and left motor cortex was found in the patients with chronic obstructive pulmonary disease (COPD) (32). Similar to COPD, airflow obstruction is also a critical symptom of asthma. Thus, we inferred that the lower variability in SMA would also be caused by oxygen desaturation related to asthma. Identical to SMA, the paracentral lobule plays an important role in the processing of sensory airway (33). A previous study reported that COPD patients showed decreased activity in the right paracentral lobule, right SMA, left fusiform gyrus, and left putamen (34), which gave powerful evidence to our study.

The left angular gyrus, as the key node of the default mode network (DMN) (35), showed an increased variability in patients in the current study. Previous studies suggested that asthmatic patients have reduced gray matter volume (36) and amplitude of low-frequency fluctuation (27) in the left angular gyrus. However, the changes of these regional abnormalities are independent (27). Thus, higher variability of left angular gyrus would also indicate low functionality, which may underlie the asthma-related emotional processing.

### GCBT-Specific Changes of Clinical Symptoms and Variability

The comparison of temporal variability between pre- and post-GCBT revealed altered variability in subcortical regions and increased variability in the left temporal pole. Rolandic operculum is related to respiratory processing (37). For example, higher FC between Rolandic operculum and right insula was found in soldiers who experienced hypoxia-reoxygenation, which suggested that the level of oxygen would lead to the changes of neuronal activity (38). This conclusion can be used to explain the altered brain functions in asthma, patients experience hypoxia during asthma attack and reoxygenation after attack. In addition, parahippocampus has a potential respiratory effect (39). For example, patients with obstructive sleep apnoea exhibited fluctuations of BOLD signal intensity in the right parahippocampus, while signal intensity was reduced after positive airway pressure (40). Thus, we inferred that the temporal variability, which was computed by BOLD signal, in the right parahippocampus would be changed accompanied with the changes of oxygen level.

The temporal variability of the Rolandic operculum and right parahippocampus were reduced after GCBT, which may reflect the therapeutic effect of GCBT for abnormal respiratory symptoms. Furthermore, abnormal variability in regions of the sensorimotor cortex were recovered after GCBT. It was consistent with previous studies, demonstrating the therapeutic mechanism of GCBT (10, 11).

### Relationship Between Variability and Clinical Symptoms

Positive correlations between ACT scores and variability values in the right caudate and left putamen were found in the current study. Caudate is a critical node associated with cognitive function, and links to the dorsolateral prefrontal cortex via the network (41, 42). It is widely recognized that mood can affect cognition (43), and negative emotional symptoms are highly prevalent in asthmatic patients. However, we did not collect the cognitive information. Meanwhile, one novel study demonstrated that increased IL-6 is negatively correlated with a lower BOLD signal in caudate (44), supporting the hypothesis that inflammation in asthma may contribute to the functional changes in caudate. Asthma control level can directly impact on asthma attack (i.e., inflammation level). Therefore, we inferred that the decreased temporal variability in the right caudate was associated with the level of inflammation.

Previous research has shown that putamen is involved in sensorimotor activity (45), suggesting an important role in gating respiratory information (46). Consistent with our study, Marchi et al. (47) reported that mean oxygen saturation during sleep is correlated with the volume of left putamen. Asthma often attacks during sleep, with a great influence on the oxygen saturation. Increased variability in the left putamen was correlated with the ACT scores, strengthening the hypothesis that asthma control level directly impacts on BOLD signal.

It should be mentioned that this study comes with a few limitations. First, the patients recruited were treated with different types of antiasthmatics, so the outcome cannot be attributed to a specific antiasthmatic. Second, the sample size studied was relatively small, the complexity of GCBT warrants larger sample sizes to explain the underpinnings more fully. Furthermore, asthma-related physiological index (e.g., inflammation and lung function) and cognitive function were not assessed in this work. However, a number of recent works have found that the global signal was highly related the brain connectivity and individual difference in behavioral phenotypes (48, 49), we did not regress the respiratory signal in this study. A combination of physiological index, cognition and temporal variability may provide better understanding of the potential mechanisms of asthma and GCBT responses.

In conclusion, we provide the evidence for the widely decreased temporal variability in asthmatic patients. Moreover, the variability in the caudate and putamen exhibited significant correlations with asthma control level. Specifically, the abnormal dynamic FC could be recovered by successful GCBT. Taken together, these findings indicated that asthma-specific changes of temporal variability may serve as promising underpinnings of GCBT for asthma.

## Data Availability Statement

The raw data supporting the conclusions of this article will be made available by the authors, without undue reservation.

## Ethics Statement

The studies involving human participants were reviewed and approved by Zhongda Hospital, Southeast University. The patients/participants provided their written informed consent to participate in this study.

## Author Contributions

YZ and YYa collected fMRI and clinical data. YZ and YK performed analysis and wrote the manuscript. YYi and ZX helped with data collection and revised the manuscript. ZH contributed to fMRI data analysis and discussion. YYu and YK designed the experiments and contributed to the manuscript revision. All authors contributed to the article and approved the submitted version.

## Conflict of Interest

The authors declare that the research was conducted in the absence of any commercial or financial relationships that could be construed as a potential conflict of interest.
